# Metabolic characterization of triple negative breast cancer

**DOI:** 10.1186/1471-2407-14-941

**Published:** 2014-12-12

**Authors:** Maria D Cao, Santosh Lamichhane, Steinar Lundgren, Anna Bofin, Hans Fjøsne, Guro F Giskeødegård, Tone F Bathen

**Affiliations:** Department of Circulation and Medical Imaging, Norwegian University of Science and Technology (NTNU), Trondheim, Norway; St. Olavs Hospital, Trondheim University Hospital, Trondheim, Norway; Department of Cancer Research and Molecular Medicine, NTNU, Trondheim, Norway; Cancer Clinic, St. Olavs Hospital, Trondheim University Hospital, Trondheim, Norway; Department of Laboratory Medicine and Children’s and Women’s Health, NTNU, Trondheim, Norway; Department of Surgery, St. Olavs Hospital, Trondheim University Hospital, Trondheim, Norway

**Keywords:** Metabolomics, HR MAS MRS, Estrogen receptor, Progesterone receptor, HER-2 receptor, Triple negative breast cancer, Choline phospholipid metabolism, Glycolysis, Glutaminolysis

## Abstract

**Background:**

The aims of this study were to characterize the metabolite profiles of triple negative breast cancer (TNBC) and to investigate the metabolite profiles associated with human epidermal growth factor receptor-2/neu (HER-2) overexpression using *ex vivo* high resolution magic angle spinning magnetic resonance spectroscopy (HR MAS MRS). Metabolic alterations caused by the different estrogen receptor (ER), progesterone receptor (PgR) and HER-2 receptor statuses were also examined. To investigate the metabolic differences between two distinct receptor groups, TNBC tumors were compared to tumors with ER^pos^/PgR^pos^/HER-2^pos^ status which for the sake of simplicity is called triple positive breast cancer (TPBC).

**Methods:**

The study included 75 breast cancer patients without known distant metastases. HR MAS MRS was performed for identification and quantification of the metabolite content in the tumors. Multivariate partial least squares discriminant analysis (PLS-DA) modeling and relative metabolite quantification were used to analyze the MR data.

**Results:**

Choline levels were found to be higher in TNBC compared to TPBC tumors, possibly related to cell proliferation and oncogenic signaling. In addition, TNBC tumors contain a lower level of Glutamine and a higher level of Glutamate compared to TPBC tumors, which indicate an increase in glutaminolysis metabolism. The development of glutamine dependent cell growth or “Glutamine addiction” has been suggested as a new therapeutic target in cancer. Our results show that the metabolite profiles associated with HER-2 overexpression may affect the metabolic characterization of TNBC. High Glycine levels were found in HER-2^pos^ tumors, which support Glycine as potential marker for tumor aggressiveness.

**Conclusions:**

Metabolic alterations caused by the individual and combined receptors involved in breast cancer progression can provide a better understanding of the biochemical changes underlying the different breast cancer subtypes. Studies are needed to validate the potential of metabolic markers as targets for personalized treatment of breast cancer subtypes.

## Background

Triple negative breast cancer (TNBC) is a heterogeneous subgroup of breast cancer characterized by the absence of expression of estrogen receptor (ER), progesterone receptor (PgR) and human epidermal growth factor receptor-2/neu (HER-2). TNBC represents approximately 15-20% of all breast cancer cases and is generally considered as the most severe subgroup of breast cancer. Patients diagnosed with TNBC are largely unresponsive to currently available targeted therapies, such as Tamoxifen and Trastuzumab, in addition to having a higher risk of relapse and a higher mortality rate compared to other breast cancer subtypes [1]. Treatment with protein inhibitors against PI3KCA and HSP90 have shown to be efficient in only a subset of TNBC [2]. Therefore, there is an urgent need to identify new molecular targets for treatment of TNBC to improve treatment care and survival of this breast cancer subgroup.

Classification of breast cancer according to molecular subtypes is highly relevant and may provide significant prognostic information related to patient outcome. Several studies have investigated the underlying genomic and transcriptomic characteristics of TNBC [3–5]. The results suggest the existence of a variety of TNBC subtypes including basal and non-basal, p53 mutated and high genomic instability, among others [3]. For example, five distinct subtypes of TNBC have been suggested based on gene expression profiles [5]. In a recent study, TNBC was subdivided into basal or 5-negative phenotype dependent on the expressions of assorted basal markers, including cytokeratin 5 (CK5) and epithelial growth factor receptor (EGFR) using immunohistochemistry (IHC) and in situ hybridization [6]. The validation of reliable markers for breast cancer sub-classification is still ongoing.

Altered energy metabolism is a new emerging hallmark of cancer [7]. Increasing evidence suggests that alterations in cancer metabolism, especially choline phospholipid and amino acid metabolism may provide potential targets for treatment of breast cancer. To our knowledge, the metabolite profiles of TNBC and the metabolic influences of HER-2 overexpression have not yet been investigated in detail. Metabolomics, defined as a systematic study of the metabolism, has proven to be an important tool for the identification of new biomarkers for targeted treatment, treatment evaluation and prediction of cancer survival [8–11]. Previous studies have shown the potential and benefit of combining the different OMICS approaches, e.g. transcriptomics and metabolomics, for better molecular characterization and stratification of breast cancer [12–15].

*Ex vivo* high resolution magic angle spinning magnetic resonance spectroscopy (HR MAS MRS) can be used for the identification and quantification of the metabolite content in a biological tissue sample. HR MAS MRS is a non-destructive technique meaning that the tissue remains intact after examination and can be used for other OMICS approaches, thus allowing for a comprehensive and detailed study of the molecular composition of the tissue. By using HR MAS MRS, more than 30 metabolites can be detected and assigned simultaneously in breast cancer tissue [16]. HR MAS MRS has been widely used to study cancer related pathways, including choline phospholipid metabolism, glycolysis (the Warburg effect), amino acids, lipids and polyamines, among others [17–19]. The metabolite profiles acquired by HR MAS MRS have shown to correlate to hormone receptor status, treatment response and survival in breast cancer [20–24].

Analysis of HR MAS MRS spectra can be challenging due to the high number of collinear variables (exceeding tens of thousands of data points per sample). Multivariate data analysis is a suitable method for analyzing the complex and high dimensional MRS data. Partial least squares discriminant analysis (PLS-DA) can be used to identify metabolic differences between distinct classes by finding linear relationships between the spectral data and class variables, e.g. receptor status [25]. In addition to multivariate modeling, quantification of the individual metabolites can be achieved by calculating the area under the peak signal.

Most studies have compared TNBC with non-triple negative breast cancer, most commonly ER^pos^/PgR^pos^ breast cancer subtype, in those studies the effects of HER-2 overexpression were not considered. In this study, we have investigated the metabolic differences between TNBC tumors and tumors with ER^pos^/PgR^pos^/HER-2^pos^ status, which for the sake of simplicity is called triple positive breast cancer (TPBC). We have also examined the influences of ER, PgR and HER-2 receptors status individually on breast cancer metabolism and explored the metabolite profiles associated with HER-2 overexpression. Metabolic alterations caused by the individual and combined hormone and growth receptors may help identify potential targets for treatment of breast cancer subtypes.

## Methods

### Patients and tumor receptor status

Included in this study were patients (n = 75) aged 34 to 90 diagnosed with breast cancer without known distant metastasis. The patients did not receive any pre-surgical therapy for their cancer disease. The biopsies were extracted immediately after surgical removal of the tumor. Parts of the tumor were used for routine analyses, including tumor grade, ER, PgR and HER-2 status (Table 1). Tumors were considered positive for ER and PgR when more than 10% of tumor cells showed positive staining by IHC. The samples were tested for HER-2 gene expression using a validated dual probe fluorescence in situ hybridization (FISH) assay (HER-2 IQFISH pharmDx/HER-2FISHpharm Dx) or for protein overexpression using a validated IHC assay (HercepTest, DAKO). The HER-2 gene was considered amplified if the gene to chromosome 17 ratio was larger than 2.0 analyzed by FISH or evidence of protein overexpression by IHC score 3+. Another part of the tumor was snap frozen immediately during surgery and stored in liquid nitrogen for MRS analysis. All patients have signed a written informed consent, and the study was approved by the Regional Ethics Committee, Central Norway.

**Table 1:** **Patient characteristics n = 75 patients**

Age (avg ± SD)		64 ± 19
Grade	I	6
	II	22
	III	30
	NA	17
Lymph node status	Pos	47
	Neg	25
	NA	3
ER	Pos	44
	Neg	31
PgR	Pos	32
	Neg	43
HER-2/neu	Pos	30
	Neg	45
TNBC		20
TPBC		11

NA: not available, ER: estrogen receptor, PgR: progesterone receptor, HER-2/neu: human epidermal growth factor receptor-2, TNBC: triple negative breast cancer, TPBC: triple positive breast cancer.

### Imprint cytology

Cytological imprint was performed to confirm the presence of tumor cells in the sample before HR MAS MRS and was used as an inclusion criterion and not as a quantitative measurement [26]. This technique is fast and requires minimal preparation. In brief, the tissue was gently pressed on a glass slide and air-dried for approximately 10 minutes. The imprints were fixed in ethanol and stained with May-Grünwald-Giemsa stain (Color-Rapid, Med-Kjemi, Norway). All imprints were reviewed by a well-trained pathologist. Samples with absence of tumor cells were excluded from further analysis.

### High resolution magic angle spinning

To minimize the effect of tissue degradation on the metabolite profiles, the samples were prepared on ice block and within a short period (5 ± 1 min). The biopsies (13 ± 3 mg) were cut to fit 30 μl disposable inserts filled with 3 μl phosphate buffered saline (PBS) in D_2_O containing 1.0 mM TSP for chemical shift referencing and 1.0 mM Format for shimming. The HR MAS spectra were acquired on a Bruker Avance DRX600 spectrometer equipped with a ^1^H/^13^C MAS probe with gradient (Bruker Biospin GmbH, Germany) using the following parameters; 5 kHz spin rate, 4°C probe temperature, cpmgpr1D sequence (Bruker Biospin GmbH, Germany) with 273.5 ms total echo time, a spectral width of 20 ppm (−5 to 15 ppm) and 256 scans (NS). For some patients, more than one biopsy (taken from different places in the tumor) were prepared and analyzed by HR MAS MRS.

### Data analysis

Following acquisition, the spectra were Fourier transformed into 65.5 k after 0.3 Hz line broadening and TSP was calibrated to 0.00 ppm (Topspin 3.1, Bruker Biospin GmbH, Germany). The following spectral preprocessing steps were carried out using Matlab R2009a (The Mathworks, Inc., USA). Spectral regions containing signals from chemical contaminations (e.g. ethanol), water, and lipids were removed before multivariate data analysis. Baseline offset was corrected by setting the lowest point of each spectrum to zero. The spectra were normalized to equal total area to account for differences in sample size. Furthermore, the spectra were peak aligned using icoshift [27]. The spectral region between 1.5 – 4.7 ppm, containing the majority of low-molecular weight metabolites, was used as the final input for the multivariate models.

PLS-DA and metabolite relative quantification were performed to evaluate the metabolic differences between the tested groups using Matlab and PLS_Toolbox 6.2.1 (Eigenvector Research, USA). The spectra were mean-centered before the PLS-DA modeling. The classification results were calculated using random cross validation (20% for testing and 80% for training, repeated 20 times). In cases where there were multiple spectra from the same patient, all of these spectra were either used for training or testing. The number of latent variables (LVs) used for all repetitions was chosen by leave one patient out cross-validation of the whole data set. Permutation testing, carried out by randomly assigning the class labels, was performed to evaluate the statistical significance of the classification results [25]. The permuted classification result was calculated as described for the PLS-DA models and repeated 1000 times. Metabolites importance in the PLS-DA loading were identified by variable importance in the projection (VIP) scores [28]. Relative metabolite quantification was performed by peak integration using mean normalized spectra after removal of water, lipids and contaminations. Statistical differences between the groups were tested by Wilcoxon testing with Benjamini Hochberg correction for multiple testing. P-values ≤ 0.05 were considered significant. The p-values adjusted for multiple testing are given as q-values. While P-values are used as an indicator of the false positives in all tested values in the dataset, the q-values are used to interpret the false discovery rate (FDR) among significant p-values. To give a more accurate indication of the FDR both p- and q-values are listed in the results. The quantification results are illustrated by heat maps (Matlab R2009a).

## Results

**Figure 1:**
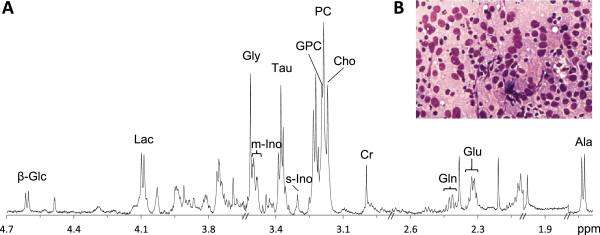
**Breast cancer metabolite spectrum and cytology image. (A)** A representative metabolite profile of breast cancer tissue acquired with HR MAS MRS. **(B)** Imprint cytology slide of breast cancer tissue stained with May-Grünwald-Giemsa staining. β-Glc: beta Glucose, Lac: Lactate, Gly: Glycine, m-Ino: myo-Inositol, Tau: Taurine, s-Ino: scyllo-Inositol, GPC: Glycerophosphocholine, PC: Phosphocholine, Cho: free Choline, Cr: Creatine, Gln: Glutamine, Glu: Glutamate, Ala: Alanine.

Spectra from biopsies with absence of tumor cells and low spectral quality with high noise and severe chemical contamination were excluded from further analysis (n = 4). In total, 106 biopsies from 73 patients were included in the data analyses. A representative metabolite spectrum of breast cancer tissue obtained by HR MAS MRS is shown in Figure 1. The metabolite data shows no significant association with tumor grade and lymph node status by PCA and PLS-DA modeling (data not shown). The PLS-DA classification results of TNBC, ER, PgR and HER-2 are summarized in Table 2.

**Table 2:** **PLS-DA classification results of TNBC, ER, PgR and HER-2 status**

	Total b/p	NEG b/p	POS b/p	CV accuracy %	CV Sensitivity %	CV Specificity %	Permutation p-value
**TNBC** ***vs*** **TPBC**	39/30	26/19	13/11	77.7	80.0	75.4	0.001
**ERneg** ***vs*** **ERpos**	106/73	41/30	65/43	72.2	76.0	68.5	<0.001
**PgRneg** ***vs*** **PgRpos**	106/73	59/42	47/31	67.8	74.0	61.6	<0.001
**HER-2neg** ***vs*** **HER-2pos**	106/73	66/43	40/30	69.1	70.2	68.0	<0.001

b = biopsies/p = patients. PLS-DA: partial least squares discriminant analysis, CV: cross validation.

### TNBC *versus*TPBC

**Figure 2:**
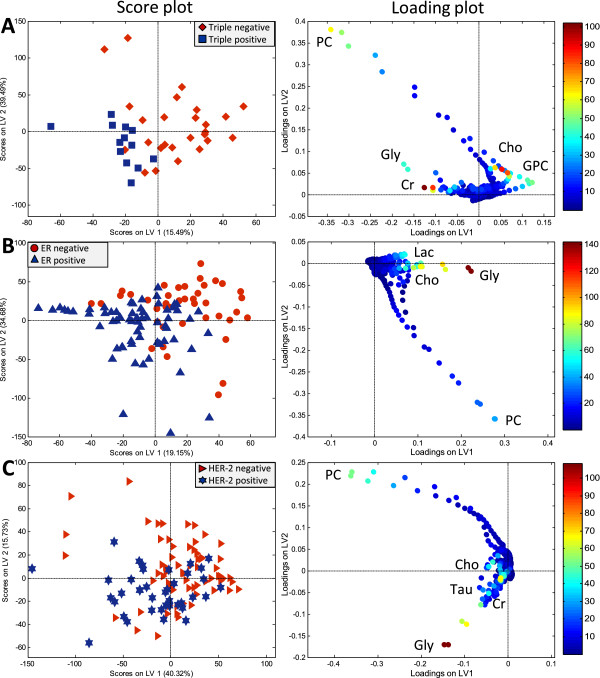
**PLS-DA score and loading plots of (A) TNBC** ***versus*** **TPBC, (B)** **ER** ^**neg**^ ***versus*** **ER** ^**pos**^ **, and (C) HER-2** ^**neg**^ ***versus*** **HER-2** ^**pos**^ **breast cancer tumors.** In the score plots (left), each symbol represents one sample. The score plots show the first and second latent variables (LV), and are used for interpreting relations between samples, thus similar samples are located close to each other. In the loading plots (right), the symbols represent metabolites that are significantly important for the discrimination between the groups. Variable importance in the projection (VIP) scores are illustrated by the heat map. The majority of TNBC, ER^neg^ and HER-2^neg^ samples have positive score for LV1. The PLS-DA model of TNBC versus TPBC shows best classification results, see Table 2. Gly: Glycine, Lac: Lactate, Cho: Choline, PC: Phosphocholine, Cr: Creatine, Tau: Taurine.

**Figure 3:**
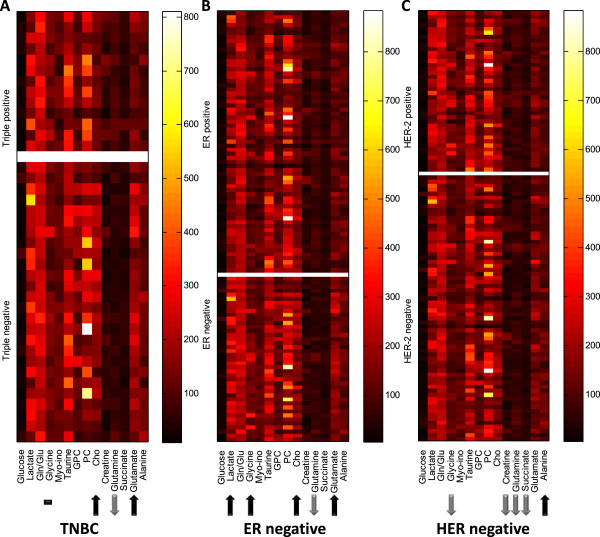
**Relative metabolite quantification of 14 metabolites and ratio illustrated by heat map.** The heat maps illustrate the metabolite intensities calculated by peak integration. The arrows show metabolites with significantly higher (↑) or lower (↓) levels in **(A)** TNBC, **(B)** ER^neg^ and **(C)** HER-2^neg^ tumors compared to TPBC, ER^pos^ and HER-2^pos^ tumors, respectively. Statistical differences between the groups were tested by Wilcoxon testing with Benjamini Hochberg correction for multiple testing.

The PLS-DA shows the highest CV accuracy for separating TNBC and TPBC (77.7%, p = 0.001). The corresponding score and loading plots show a clear separation between the two groups. TNBC is characterized with higher levels of Choline and Glycerophosphocholine (GPC), and a lower level of Creatine compared to TPBC (Figure 2A). Based on the loadings, high levels of PC and Glycine were observed in some tumors, but their influence in the classification model are unclear. Relative quantification shows consistently higher levels of Choline (p = 0.008, q = 0.041) in TNBC tumors. Lower levels of Glutamine (p < 0.001, q = 0.001) and higher levels of Glutamate (p = 0.002, q = 0.015) were also observed in TNBC compared to TPBC tumors (Figure 3A). Creatine appears to be important for separating TNBC and TPBC in the multivariate analysis identified by a high value of VIP score. Lower levels of Creatine were also found in TNBC compared to TPBC tumors by relative quantification, however, the q-value was not significant (p-value = 0.031, q-value = *0.109*).

### Hormone receptor status

PLS-DA models show clear separations between ER^neg^ and ER^pos^ (72.2%, p < 0.001), and PgR^neg^ and PgR^pos^ (67.8%, p < 0.001) tumors. ER^neg^ tumors show higher levels of Glycine, Choline, and Lactate compared to ER^pos^ tumors, as shown in the score and loading plots (Figure 2B). According to the VIP scores, Glycine appears to be most important for the discrimination between ER^neg^ and ER^pos^. Higher levels of Glycine (p = 0.002, q = 0.010), Choline (p = 0.021, q = *0.067*), Lactate (p < 0.000, q = 0.001), and Glutamate (p <0.001, q <0.001) and lower level of Glutamine (p <0.001, q <0.001) were observed in ER^neg^ compared to ER^pos^ tumors by relative quantification (Figure 3B). PC levels appear to be high in some tumors from both groups, and could not be used to discriminate between ER^neg^ and ER^pos^ tumors. PLS-DA classification and relative quantification of PgR^neg^ and PgR^pos^ tumors show similar metabolite profiles as ER^neg^ and ER^pos^ tumors (data not shown).

### HER-2 status

HER-2^neg^ and HER-2^pos^ tumors were discriminated by PLS-DA with 69.1% CV accuracy (p < 0.001). Contrary to ER^neg^ and PgR^neg^, HER-2^neg^ tumors have a lower level of Glycine (p = 0.002, q = 0.012) compared to HER-2^pos^ tumors (Figures 2C and 3C). Similar to what was observed for ER^neg^ and PgR^neg^ tumors, lower levels of Glutamine (p = 0.003, q = 0.017) were observed in HER-2^neg^ compared to HER-2^pos^ tumors detected by relative quantification. In addition to the changes in Glycine and Glutamine, HER-2^neg^ tumors also display higher levels of Alanine (p = 0.010, q = 0.039), and lower levels of Succinate (p = 0.001, q = 0.012) and Creatine (p = 0.024, q *= 0.075*) compared to HER-2^pos^ tumors by relative quantification. In the loading plot, PC levels appear to be higher in some HER-2^neg^ compared to HER-2^pos^ tumors. However, the relative quantification result shows no significant difference in PC levels between the two groups.

### HER-2 metabolite profiles in tumors with different ER and PgR status

**Figure 4:**
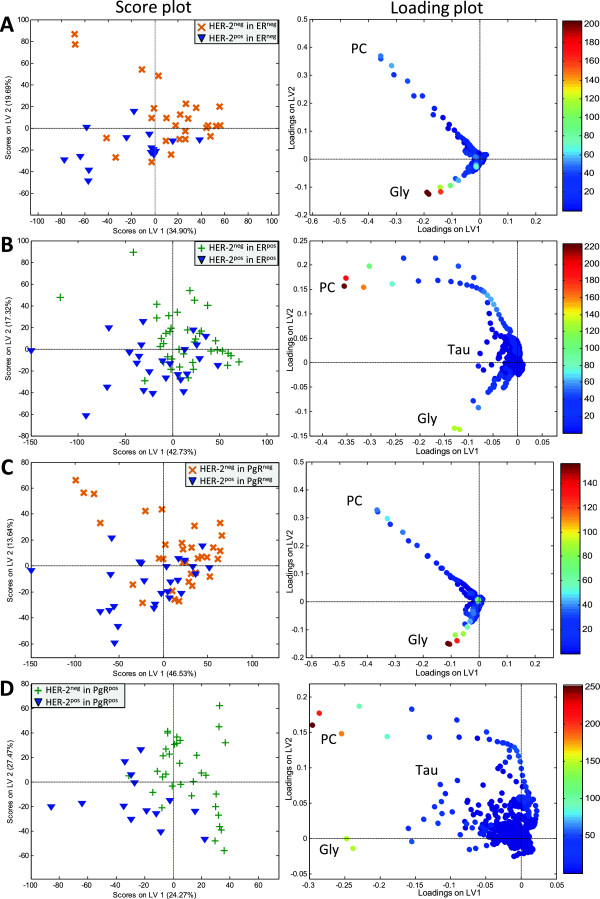
**PLS-DA score and loading plots of HER-2** ^**neg**^ ***versus*** **HER-2** ^**pos**^ **status in (A) ER** ^**neg**^ **, (B) ER** ^**pos**^ **, (C) PgR** ^**neg**^ **, and (D) PgR** ^**pos**^ **tumors.** The score plots show the first and second latent variables (LV). In the loading plot, VIP scores are illustrated by heat map. The majority of HER-2^neg^ tumors show positive score for LV1, while most HER-2^pos^ tumors show negative score for LV1. HER-2^pos^ tumors contain higher Glycine level compared to HER-2^neg^ tumors. LV: latent variable, Gly: Glycine. PC: Phosphocholine.

To investigate the metabolic influences of HER-2 status independently of the hormone receptors status, the metabolite profiles associated with HER-2 status were examined within ER^neg^, ER^pos^, PgR^neg^, and PgR^pos^ tumors separately. The PLS-DA results are shown in Table 3. The scores and loadings of PLS-DA models show higher levels of Glycine in HER-2^pos^ compared to HER-2^neg^ tumors irrespective of ER and PgR status (Figure 4A-D). Glycine levels determined by relative quantification showed a trend of higher levels in HER-2^pos^ compared to HER-2^neg^ tumors in the different ER and PgR status groups (p < 0.021 and q < *0.133*). Glutamine also showed a trend of higher level in HER-2^pos^ compared to HER-2^neg^ tumors (p < 0.034 and q < *0.179*). In the PLS-DA models, PC appears to be high in some HER-2^neg^ tumors. However, PC level was not significantly different between HER-2^pos^ and HER-2^neg^ by relative quantification.

**Table 3:** **PLS-DA classification results of HER-2 status in tumors with different ER and PgR status**

	Total b/p	HER-2neg b/p	HER-2pos b/p	CV accuracy %	CV Sensitivity %	CV Specificity %	Permutation p-value
**HER-2neg** ***vs*** **HER-2pos in ERneg**	41/30	26/19	15/11	70.1	63.3	76.8	0.013
**HER-2neg** ***vs*** **HER-2pos in ERpos**	65/43	40/24	25/19	66.1	67.9	64.3	0.017
**HER-2neg** ***vs*** **HER-2pos in PgRneg**	59/42	33/24	26/18	68.8	65.3	72.3	0.006
**HER-2neg** ***vs*** **HER-2pos in PgRpos**	47/31	33/19	14/12	70.1	68.3	71.8	0.014

b = biopsies/p = patients. PLS-DA: partial least squares discriminant analysis, CV: cross validation.

## Discussion

Triple negative breast cancer is characterized as being estrogen receptor, progesterone receptor and HER-2/neu receptor negative; it is a heterogeneous breast cancer subtype that is difficult to treat and is associated with high recurrence and poor outcome [1]. Several studies have investigated the underlying genomic and gene expression patterns of TNBC [2–5] while the metabolite profiles of TNBC have not yet been investigated in detail. Most studies have compared TNBC with ER^pos^/PgR^pos^ breast cancer subtype, which does not take into consideration the influence of HER-2 status on breast cancer molecular profiles. In this study we investigated the metabolite profiles of patients with TNBC compared to TPBC and showed that these two groups could be successfully separated based on the metabolite profiles of tissue biopsies. In accordance with previous studies, altered metabolite profiles were observed in tumors with different expression of ER and PgR [21]. Furthermore, our results show that overexpression of HER-2 might cause alterations to the metabolite profiles of breast cancer independent of hormone receptor status, thus affecting the differentiation between TNBC and TPBC.

The basal-like breast cancer subtype is defined through gene expression profiling and is considered to be a more aggressive breast cancer subtype compared to luminal-like and HER-2 enriched gene expression subtypes. The majority of basal-like tumors are TNBC, but not all TNBC are defined as basal-like by gene expression. As previously published, the discrepancy rate is approximately 20–30 % [29]. Furthermore, there exists a significant overlap between TNBC, basal-like and BRCA-1 breast cancer [30]. In our study, TNBC has significantly higher Choline levels compared to TPBC, and this is in accordance with previous findings where higher Choline levels were detected in the more aggressive basal-like xenografts and TNBC patients as compared to the less aggressive luminal-like xenografts and ER^pos^/PgR^pos^ breast cancer patients [12]. In another study, a significantly higher total Choline (tCho = PC + GPC + Choline) signal to noise ratio (tCho/SNR) was detected in TNBC when compared to non-triple negative tumors using *in vivo* MRS [31]. Choline-containing metabolites are involved in cell signaling, lipid metabolism, and cell membrane synthesis and degradation. The tCho level detected by *in vivo* and *ex vivo* MRS has been suggested as a biomarker for breast cancer diagnosis and response to chemotherapy [19].

Patients with basal-like breast cancer have been shown to be more sensitive to anthracycline-based neoadjuvant chemotherapy than the luminal subtype and a higher percentage of patients with a pathological complete response (pCR) to the treatment was achieved in the basal-like compared to luminal subtypes [32]. However, for patients with residual disease after chemotherapy, the basal-like subtypes showed worse overall survival than the luminal-like. These results indicate that chemotherapy alone is not sufficient to treat TNBC and that more advanced targeted therapy is needed to improve the prognosis of this patient subgroup. Moreover, assessment of clinical response (i.e. changes in tumor size) alone might not be a good predictive measure for treatment, as it cannot give information about the molecular state of the tumor. Interestingly, decreased levels of choline-containing compounds in response to neoadjuvant chemotherapy have been detected in patients with better survival rate [22, 23]. Targeting the genes and enzymes involved in the choline phospholipid metabolism is currently under investigation, and so far the results have been promising. Down-regulation of choline kinase alpha (CHKA), the gene regulating the conversion of Choline to PC, has been shown to decrease cell proliferation, and to increase the effect of chemotherapy in ovarian [33] and breast cancers [34], whereas CHKA overexpression increases drug resistance in breast cancer cells [35]. The CHKA inhibitor is currently under phase I clinical trial. Our results suggest that targeting the genes/enzymes responsible for the choline phospholipid metabolism may provide new molecular targets for treatment of TNBC.

Alterations in ER, PgR and HER-2 expression have proven to play a major role in breast cancer progression, with ER^pos^ and PgR^pos^ tumors having better prognosis, while HER-2 overexpression is associated with a worse prognosis. Thus metabolic alterations caused by these hormone and growth receptors are highly relevant, especially because the molecular reasons behind their overexpression/amplification remain largely unknown. Similar metabolite profiles were observed in tumors with ER^neg^ and PgR^neg^, and ER^pos^ and PgR^pos^ status. In accordance with previous findings, we found a higher level of Glycine in patients with ER^neg^ and PgR^neg^ tumors compared to ER^pos^ and PgR^pos^, respectively [21]. Higher levels of Choline and Lactate were also observed in ER^neg^ and PgR^neg^ tumors, which suggest enhanced glycolytic activity and tumor aggressiveness. ER status is generally accepted as an independent prognostic and predictive factor, while the significance of PgR status is less clear [36].

Although TNBC is considered to be a more aggressive breast cancer subgroup due to low response to available treatment, the overexpression of HER-2 itself is associated with poorer prognosis compared to HER-2 negativity [37]. Patients identified with HER-2^pos^ tumors are often treated with Trastuzumab. Noticeably, it has been reported that about 20-30% of HER-2^pos^ patients fail to respond to first time treatment with Trastuzumab and about 15% of patients will develop resistance to this drug [38, 39]. Therefore, there is also a need to identify new molecular targets for treatment of this breast cancer subtype. In our study, we found high levels of Glycine and Alanine to be associated with HER-2^pos^ breast tumors. Alanine is involved in the synthesis of Glycine from Pyruvate and Serine. High levels of Glycine have previously been shown to correlate with poor prognosis in breast cancer [23, 40, 41]. Glycine is an amino acid involved in the synthesis of proteins, nucleotides and glutathione. The potential role of Glycine as a tumor biomarker has also been studied in human brain tumors, where it was found to positively correlate with tumor grade [42, 43]. Moreover, the synthesis of Glycine from Glucose has been shown to correlate with rapid cancer cell proliferation [44].

Interestingly, we found higher levels of Glycine to be associated with HER-2 overexpression in the ER^neg^, ER^pos^, PgR^neg^, and PgR^pos^ tumors separately, which suggest Glycine to be a specific marker for HER-2 amplification regardless of the ER and PgR status. ER^neg^ and PgR^neg^ tumors and ER^pos^ and PgR^pos^ tumors show comparable results. Tumors overexpressing HER-2 have shown to acquire resistance to estrogen therapy which suggests that there exists a crosstalk between ER and HER-2 status [45]. The p-values for differences in Glycine relative concentration between HER-2^pos^ and HER-2^neg^ were significant before multiple corrections, while the adjusted p-values showed trends towards significance. However, the chance of false positive result as described by the false discovery rate was low. In our cohort, we could not detect any differences in Glycine between TNBC and TPBC, possibly due to the high level of Glycine in ER^neg^ and PgR^neg^ and low level of Glycine in HER-2^neg^ tumors, which may cancel out the differences in Glycine between TNBC and TPBC. Based on our results we suggest Glycine to be associated with tumor aggressiveness in HER-2^pos^ breast cancer. Recently, there has been an increasing interest in detecting the circulating HER-2 protein in serum samples for use as a complementary assay to IHC and FISH analysis for diagnosis, but also for use as a prognostic marker for breast cancer recurrence [46, 47]. High throughput screening of serum metabolites, including Glycine, is feasible using MRS or other laboratory assays and should be investigated further as a breast cancer prognostic marker.

Furthermore, significant changes in other amino acids were also observed between the different breast cancer subtypes. Tumors negative for ER and PgR, and TNBC tumors contain lower levels of Glutamine and higher levels of Glutamate compared to tumors with positive receptor statuses which might result from increased glutaminolysis metabolism. Glutamine plays an important role in nucleotide and protein synthesis and in mitochondrial energy metabolism. Increased uptake and metabolism of Glutamine through glutaminolysis can provide a proliferating cell with significant amount of NADPH requirement [48]. Some cancer cells develop addiction to Glutamine and become dependent on Glutamine to support cell growth and activation of signaling molecules, e.g. mTOR kinase [49]. Recent studies have explored the potential of targeting amino-oxyacetic acid (AOA) for inhibition of cell proliferation in breast cancer xenograft models [50]. In a recent study, the expression of glutamine-related proteins was found to be highest in HER-2 subtypes compared to other breast cancer subtypes [51]. In our study, we found a higher level of Glutamine in HER-2^pos^ compared to HER-2^neg^ tumors. The role of Glutamine metabolism in breast cancer prognosis and treatment is still under investigation. However, increasing evidence suggests that alterations in cancer metabolism, especially the choline phospholipid and amino acid metabolisms may provide potential targets for treatment of breast cancer.

In 2010, the American Society of Clinical Oncology (ASCO) and College of American Pathologists (CAP) issued guidelines that recommended the threshold for determining ER and PgR positivity to be decreased from 10% to 1%, in order to standardize the determination of hormone receptor status by IHC and also to increase the number of patients eligible for hormone therapy. In this study, the 10% cutoff value was used according to The Norwegian Breast Cancer Group recommendation at the time of inclusion. There is an ongoing debate about whether the decrease in ER and PgR threshold has led to a group of false ER^pos^ tumors. Studies have shown that the majority of low ER^pos^ tumors (≥1 < 10%) were identified as basal-like or HER-2 enriched tumors with pathological features more similar to ER^neg^ than ER^pos^ tumors, while only a minority of low ER^neg^ tumors was classified as luminal A subtype [52–54]. In a large breast cancer study by Engstrøm et al., only 24 out of 909 cases (2.7%) showed positive staining for ER in ≥1 < 10% of the tumor cells and were classified as ER^pos^ according to the new guidelines [6]. The authors found little or no change in the Kaplan–Meier and Cox results when comparing the new 1% cut-off with the previously 10% cut-off in their study.

In our cohort, we found no correlation between the metabolite profiles and tumor grade and also no correlation between the metabolite profiles and lymph node status. We have previously investigated nodal metastasis using metabolite data from the primary tumor using PLS-DA, and the results showed only a weak correlation with nodal spread [21]. Based on our results, the differences in the metabolite profiles observed are indeed resulting from the hormone and growth receptor status and not dependent on tumor grade or nodal metastasis.

This study is restricted by some limitations including the small number of samples in each subgroup and the lack of normal control tissues. Most of the patients in this study were recruited less than 5 years ago, long term follow-up is thus yet not available, however this aspect will be important in future studies. In addition, it would be interesting to investigate if Ki67 overexpression is associated with adverse metabolic profiles; Ki67 was not included, however, as part of the standard histochemical staining at the time of patient recruitment in our study. Statins are a class of drugs that reduce the production of cholesterol by inhibiting the enzyme HMG-CoA reductase. In a recent study, treatment with Lovastatin was shown to decrease choline-containing phospholipids and inhibit the proliferation in breast cancer cells *in vitro* [55]. The effects of statins on the metabolite profiles should also be investigated in more details.

Breast cancer tumor heterogeneity is a common challenge. To minimize the effect of heterogeneity, we have chosen to include only tumors with T1/T2 stages (<5 cm in diameter) without distant metastasis. In this study, the effect of heterogeneity on the metabolic profile when sampling multiple biopsies was tested by comparing the average correlation of 35 random pairs repeated 1000 times *versus* 35 pairs of sample from the same patient. Our results show that the variation between patients is significantly higher than the variation within a patient (p-value < 0.001).

## Conclusion

The classification of TNBC and TPBC tumors were successfully separated based on the metabolite profiles. Choline levels were found to be higher in TNBC compared to TPBC, possibly related to tumor proliferation and oncogenic signaling. TNBC tumors had a lower level of Glutamine and a higher level of Glutamate compared to TPBC tumors, which indicates an increase in glutaminolysis metabolism and suggests the development of glutamine dependent cell growth. The classification of ER, PgR and HER-2 status were also successful. We found significantly higher levels of Glycine in HER-2^pos^ breast cancer, which supports the potential of Glycine as a marker for tumor aggressiveness. Further studies are needed to validate the potential of metabolic markers as targets for personalized treatment of breast cancer subtypes.

## Abbreviations

AOA: Amino-oxyacetic acid
CHKA: Choline kinase alpha
CK5: Cytokeratin 5
EGFR: Epithelial growth factor receptor
ER: Estrogen receptor
FDR: False discovery rate
FISH: Fluorescence in situ hybridization
GPC: Glycerophosphocholine
HER-2: Human epidermal growth factor receptor-2/neu
HR MAS MRS: High resolution magic angle spinning magnetic resonance spectroscopy
LV: Latent variables
NS: Number of scans
PBS: Phosphate buffered saline
PC: Phosphocholine
pCR: Pathological complete response
PgR: Progesterone receptor
PLS-DA: Partial least squares discriminant analysis
SNR: Signal to noise ratio
tCho: Total choline
TNBC: Triple negative breast cancer
TPBC: Triple positive breast cancer
VIP: Variable importance in the projection.

## References

[1] Kaplan HG, Malmgren JA (2008). Impact of triple negative phenotype on breast cancer prognosis. Breast J.

[2] Hirshfield KM, Ganesan S (2013). Triple-negative breast cancer: molecular subtypes and targeted therapy. Curr Opin Obstet Gynecol.

[3] Xu H, Eirew P, Mullaly SC, Aparicio S (2014). The omics of triple-negative breast cancers. Clin Chem.

[4] Stevens KN, Vachon CM, Couch FJ (2013). Genetic susceptibility to triple-negative breast cancer. Cancer Res.

[5] Kreike B, van Kouwenhove M, Horlings H, Weigelt B, Peterse H, Bartelink H, van de Vijver MJ (2007). Gene expression profiling and histopathological characterization of triple-negative/basal-like breast carcinomas. Breast Cancer Res.

[6] Engstrom MJ, Opdahl S, Hagen AI, Romundstad PR, Akslen LA, Haugen OA, Vatten LJ, Bofin AM (2013). Molecular subtypes, histopathological grade and survival in a historic cohort of breast cancer patients. Breast Cancer Res Treat.

[7] Hanahan D, Weinberg Robert A (2011). Hallmarks of cancer: the next generation. Cell.

[8] Nordstrom A, Lewensohn R (2010). Metabolomics: moving to the clinic. J Neuroimmune Pharmacol.

[9] Bathen TF, Geurts B, Sitter B, Fjosne HE, Lundgren S, Buydens LM, Gribbestad IS, Postma G, Giskeodegard GF (2013). Feasibility of MR metabolomics for immediate analysis of resection margins during breast cancer surgery. PLoS One.

[10] Claudino WM, Goncalves PH, di Leo A, Philip PA, Sarkar FH (2012). Metabolomics in cancer: a bench-to-bedside intersection. Crit Rev Oncol Hematol.

[11] Aboud OA, Weiss RH (2013). New opportunities from the cancer metabolome. Clin Chem.

[12] Moestue SA, Borgan E, Huuse EM, Lindholm EM, Sitter B, Borresen-Dale AL, Engebraaten O, Maelandsmo GM, Gribbestad IS (2010). Distinct choline metabolic profiles are associated with differences in gene expression for basal-like and luminal-like breast cancer xenograft models. BMC Cancer.

[13] Borgan E, Sitter B, Lingjaerde OC, Johnsen H, Lundgren S, Bathen TF, Sorlie T, Borresen-Dale AL, Gribbestad IS (2010). Merging transcriptomics and metabolomics–advances in breast cancer profiling. BMC Cancer.

[14] Cuperlovic-Culf M, Chute IC, Culf AS, Touaibia M, Ghosh A, Griffiths S, Tulpan D, Leger S, Belkaid A, Surette ME, Ouellette RJ (2011). 1H NMR metabolomics combined with gene expression analysis for the determination of major metabolic differences between subtypes of breast cell lines. Chem Sci.

[15] Martinez-Outschoorn UE, Prisco M, Ertel A, Tsirigos A, Lin Z, Pavlides S, Wang C, Flomenberg N, Knudsen ES, Howell A, Pestell RG, Sotgia F, Lisanti MP (2011). Ketones and lactate increase cancer cell “stemness”, driving recurrence, metastasis and poor clinical outcome in breast cancer: achieving personalized medicine via Metabolo-Genomics. Cell Cycle.

[16] Sitter B, Sonnewald U, Spraul M, Fjosne HE, Gribbestad IS (2002). High-resolution magic angle spinning MRS of breast cancer tissue. NMR Biomed.

[17] Bertilsson H, Tessem MB, Flatberg A, Viset T, Gribbestad I, Angelsen A, Halgunset J (2012). Changes in gene transcription underlying the aberrant citrate and choline metabolism in human prostate cancer samples. Clin Cancer Res.

[18] Giskeodegard GF, Bertilsson H, Selnaes KM, Wright AJ, Bathen TF, Viset T, Halgunset J, Angelsen A, Gribbestad IS, Tessem MB (2013). Spermine and citrate as metabolic biomarkers for assessing prostate cancer aggressiveness. PLoS One.

[19] Glunde K, Bhujwalla ZM, Ronen SM (2011). Choline metabolism in malignant transformation. Nat Rev Cancer.

[20] Bathen TF, Jensen LR, Sitter B, Fjosne HE, Halgunset J, Axelson DE, Gribbestad IS, Lundgren S (2007). MR-determined metabolic phenotype of breast cancer in prediction of lymphatic spread, grade, and hormone status. Breast Cancer Res Treat.

[21] Giskeodegard GF, Grinde MT, Sitter B, Axelson DE, Lundgren S, Fjosne HE, Dahl S, Gribbestad IS, Bathen TF (2010). Multivariate modeling and prediction of breast cancer prognostic factors using MR metabolomics. J Proteome Res.

[22] Cao MD, Sitter B, Bathen TF, Bofin A, Lonning PE, Lundgren S, Gribbestad IS (2011). Predicting long-term survival and treatment response in breast cancer patients receiving neoadjuvant chemotherapy by MR metabolic profiling. NMR Biomed.

[23] Cao MD, Giskeodegard GF, Bathen TF, Sitter B, Bofin A, Lonning PE, Lundgren S, Gribbestad IS (2012). Prognostic value of metabolic response in breast cancer patients receiving neoadjuvant chemotherapy. BMC Cancer.

[24] Sitter B, Lundgren S, Bathen TF, Halgunset J, Fjosne HE, Gribbestad IS (2006). Comparison of HR MAS MR spectroscopic profiles of breast cancer tissue with clinical parameters. NMR Biomed.

[25] Westerhuis J, Hoefsloot H, Smit S, Vis D, Smilde A, van Velzen E, van Duijnhoven J, van Dorsten F (2008). Assessment of PLSDA cross validation. Metabolomics.

[26] Mangia A, Chiriatti A, Chiarappa P, Incalza MA, Antonaci G, Pilato B, Simone G, Tommasi S, Paradiso A (2008). Touch imprint cytology in tumor tissue banks for the confirmation of neoplastic cellularity and for DNA extraction. Arch Pathol Lab Med.

[27] Savorani F, Tomasi G, Engelsen SB (2010). icoshift: a versatile tool for the rapid alignment of 1D NMR spectra. J Magn Reson.

[28] Chong I-G, Jun C-H (2005). Performance of some variable selection methods when multicollinearity is present. Chemometr Intell Lab Syst.

[29] Prat A, Perou CM (2011). Deconstructing the molecular portraits of breast cancer. Mol Oncol.

[30] Oakman C, Viale G, Di Leo A (2010). Management of triple negative breast cancer. Breast.

[31] Shin HJ, Baek HM, Cha JH, Kim HH (2012). Evaluation of breast cancer using proton MR spectroscopy: total choline peak integral and signal-to-noise ratio as prognostic indicators. AJR Am J Roentgenol.

[32] Carey LA, Dees EC, Sawyer L, Gatti L, Moore DT, Collichio F, Ollila DW, Sartor CI, Graham ML, Perou CM (2007). The triple negative paradox: primary tumor chemosensitivity of breast cancer subtypes. Clin Cancer Res.

[33] Granata A, Nicoletti R, Tinaglia V, De Cecco L, Pisanu ME, Ricci A, Podo F, Canevari S, Iorio E, Bagnoli M, Mezzanzanica D (2013). Choline kinase-alpha by regulating cell aggressiveness and drug sensitivity is a potential druggable target for ovarian cancer. Br J Cancer.

[34] Mori N, Glunde K, Takagi T, Raman V, Bhujwalla ZM (2007). Choline kinase down-regulation increases the effect of 5-fluorouracil in breast cancer cells. Cancer Res.

[35] Shah T, Wildes F, Penet MF, Winnard PT, Glunde K, Artemov D, Ackerstaff E, Gimi B, Kakkad S, Raman V, Bhujwalla ZM (2010). Choline kinase overexpression increases invasiveness and drug resistance of human breast cancer cells. NMR Biomed.

[36] Hefti MM, Hu R, Knoblauch NW, Collins LC, Haibe-Kains B, Tamimi RM, Beck AH (2013). Estrogen receptor negative/progesterone receptor positive breast cancer is not a reproducible subtype. Breast Cancer Res.

[37] Rubin I, Yarden Y (2001). The basic biology of HER2. Ann Oncol.

[38] Spector NL, Blackwell KL (2009). Understanding the mechanisms behind trastuzumab therapy for human epidermal growth factor receptor 2-positive breast cancer. J Clin Oncol.

[39] Nahta R, Shabaya S, Ozbay T, Rowe DL (2009). Personalizing HER2-targeted therapy in metastatic breast cancer beyond HER2 status: what we have learned from clinical specimens. Curr Pharmacogenomics Person Med.

[40] Giskeodegard GF, Lundgren S, Sitter B, Fjosne HE, Postma G, Buydens LM, Gribbestad IS, Bathen TF (2012). Lactate and glycine-potential MR biomarkers of prognosis in estrogen receptor-positive breast cancers. NMR Biomed.

[41] Sitter B, Bathen TF, Singstad TE, Fjosne HE, Lundgren S, Halgunset J, Gribbestad IS (2010). Quantification of metabolites in breast cancer patients with different clinical prognosis using HR MAS MR spectroscopy. NMR Biomed.

[42] Davies NP, Wilson M, Natarajan K, Sun Y, MacPherson L, Brundler MA, Arvanitis TN, Grundy RG, Peet AC (2010). Non-invasive detection of glycine as a biomarker of malignancy in childhood brain tumours using in-vivo 1H MRS at 1.5 tesla confirmed by ex-vivo high-resolution magic-angle spinning NMR. NMR Biomed.

[43] Righi V, Andronesi OC, Mintzopoulos D, Black PM, Tzika AA (2010). High-resolution magic angle spinning magnetic resonance spectroscopy detects glycine as a biomarker in brain tumors. Int J Oncol.

[44] Jain M, Nilsson R, Sharma S, Madhusudhan N, Kitami T, Souza AL, Kafri R, Kirschner MW, Clish CB, Mootha VK (2012). Metabolite profiling identifies a key role for glycine in rapid cancer cell proliferation. Science.

[45] Osborne CK, Shou J, Massarweh S, Schiff R (2005). Crosstalk between estrogen receptor and growth factor receptor pathways as a cause for endocrine therapy resistance in breast cancer. Clin Cancer Res.

[46] Esteva FJ, Cheli CD, Fritsche H, Fornier M, Slamon D, Thiel RP, Luftner D, Ghani F (2005). Clinical utility of serum HER2/neu in monitoring and prediction of progression-free survival in metastatic breast cancer patients treated with trastuzumab-based therapies. Breast Cancer Res.

[47] Di Gioia D, Dresse M, Mayr D, Nagel D, Heinemann V, Kahlert S, Stieber P (2014). Serum HER2 supports HER2-testing in tissue at the time of primary diagnosis of breast cancer. Clin Chim Acta.

[48] Wise DR, Thompson CB (2010). Glutamine addiction: a new therapeutic target in cancer. Trends Biochem Sci.

[49] Nicklin P, Bergman P, Zhang B, Triantafellow E, Wang H, Nyfeler B, Yang H, Hild M, Kung C, Wilson C, Myer VE, MacKeigan JP, Porter JA, Wang YK, Cantley LC, Finan PM, Murphy LO (2009). Bidirectional transport of amino acids regulates mTOR and autophagy. Cell.

[50] Thornburg JM, Nelson KK, Clem BF, Lane AN, Arumugam S, Simmons A, Eaton JW, Telang S, Chesney J (2008). Targeting aspartate aminotransferase in breast cancer. Breast Cancer Res.

[51] Kim S, Kim do H, Jung WH, Koo JS (2013). Expression of glutamine metabolism-related proteins according to molecular subtype of breast cancer. Endocr Relat Cancer.

[52] Prabhu JS, Korlimarla A, Desai K, Alexander A, Raghavan R, Anupama C, Dendukuri N, Manjunath S, Correa M, Raman N, Kalamdani A, Prasad M, Gopinath KS, Srinath BS, Sridhar TS (2014). A majority of low (1-10%) ER positive breast cancers behave like hormone receptor negative tumors. J Cancer.

[53] Deyarmin B, Kane JL, Valente AL, van Laar R, Gallagher C, Shriver CD, Ellsworth RE (2013). Effect of ASCO/CAP guidelines for determining ER status on molecular subtype. Ann Surg Oncol.

[54] Iwamoto T, Booser D, Valero V, Murray JL, Koenig K, Esteva FJ, Ueno NT, Zhang J, Shi W, Qi Y, Matsuoka J, Yang EJ, Hortobagyi GN, Hatzis C, Symmans WF, Pusztai L (2012). Estrogen receptor (ER) mRNA and ER-related gene expression in breast cancers that are 1% to 10% ER-positive by immunohistochemistry. J Clin Oncol.

[55] Klawitter J, Shokati T, Moll V, Christians U, Klawitter J (2010). Effects of lovastatin on breast cancer cells: a proteo-metabonomic study. Breast Cancer Res.

[CR56] The pre-publication history for this paper can be accessed here:http://www.biomedcentral.com/1471-2407/14/941/prepub

